# Photophysical Properties of 1,3-Diphenylisobenzofuran as a Sensitizer and Its Reaction with O_2_

**DOI:** 10.3390/molecules30143021

**Published:** 2025-07-18

**Authors:** Ștefan Stan, João P. Prates Ramalho, Alexandru Holca, Vasile Chiș

**Affiliations:** 1 Faculty of Physics, Babes-Bolyai University, Str. M. Kogălniceanu 1, RO-400084 Cluj-Napoca, Romania; stefan.stan@ubbcluj.ro (Ș.S.); alexandru.holca@ubbcluj.ro (A.H.); 2 Department of Chemistry and Biochemistry, School of Science and Technology, University of Évora, Rua Romão Ramalho 59, 7000-671 Évora, Portugal; jpcar@uevora.pt; 3 Hercules Laboratory, University of Évora, Palácio do Vimioso, Largo Marquês de Marialva 8, 7000-809 Évora, Portugal; 4 Nanobiophotonics and Laser Microspectroscopy Center, Interdisciplinary Research Institute in Bio-Nano-Sciences, Babes-Bolyai University, T. Laurian 42, RO-400271 Cluj-Napoca, Romania

**Keywords:** 1,3-diphenylisobenzofuran, TD-DFT, singlet oxygen detection, photophysical properties, computational spectroscopy, intrinsic reaction coordinate (IRC)

## Abstract

1,3-Diphenylisobenzofuran (DPBF) is a widely used fluorescent probe for singlet oxygen (^1^O_2_) detection in photodynamic applications. In this work, we present an integrated experimental and computational analysis to describe its spectroscopic, photophysical, and reactive properties in ethanol, DMSO, and DMF. UV-Vis and fluorescence measurements across a wide concentration range show well-resolved S_0_ → S_1_ electronic transition of a π → π* nature with small red shifts in polar aprotic solvents. Fluorescence lifetimes increase slightly with solvent polarity, showing stabilization of the excited state. The 2D PES and Boltzmann populations analysis indicate two co-existing conformers (C_s_ and C_2_), with C_s_ being slightly more stable at room temperature. TD-DFT calculations have been performed using several density functionals and the 6-311+G(2d,p) basis set to calculate absorption/emission wavelengths, oscillator strengths, transition dipole moments, and radiative lifetimes. Overall, cam-B3LYP and ωB97X-D provided the best agreement with experiments for the photophysical data across all solvents. The photophysical behavior of DPBF upon interaction with ^1^O_2_ can be explained by a small-barrier, two-step reaction pathway that goes through a zwitterionic intermediate, resulting in the formation of 2,5-endoperoxide. This work explains the photophysical properties and reactivity of DPBF, therefore providing a solid basis for future studies involving singlet oxygen.

## 1. Introduction

1,3-Diphenylisobenzofuran (DPBF), with the chemical formula C_20_H_14_O, is a popular scavenger for singlet oxygen (^1^O_2_) in the electronic state ^1^Δ_g_ and stands out as a widely used fluorescent probe due to its high reactivity with singlet oxygen and the unique spectroscopic properties of the resulting products. Following interaction with ^1^O_2_, DPBF undergoes a [4+2] cycloaddition to form an endoperoxide, which subsequently decomposes into 1,2-dibenzoylbenzene, a non-fluorescent compound [1,2]. The molecule strongly absorbs electromagnetic radiation at a wavelength of 410 nm and emits fluorescence radiation with a maximum centered around the 450–480 nm range [3].

Although DPBF is widely used, it shows limited specificity in complex systems. In addition to singlet oxygen, 1,3-diphenylisobenzofuran has been shown to react with a variety of reactive species like hydroxyl ($H\dot{O}$), alkoxyl ($R\dot{O}$), peroxyl ($RO\dot{O}$), and carbon-centered radicals, as well as reactive nitrogen species (RNO) such as nitrogen dioxide (NO_2_) and peroxynitrite (ONOO^−^). This can lead to the formation of similar non-fluorescent products, which can complicate the interpretation of results in biological systems [2,4].

Furthermore, DPBF shows strong solvent dependence, and it should be carefully considered when designing accurate singlet oxygen detection methods, as the solvent can affect both the stability and spectroscopic behavior of DPBF [3].

More recently, DPBF has been successfully integrated into nanostructured theranostic platforms for the real-time monitoring of singlet oxygen generation during PDT, enabling simultaneous generation and detection of ^1^O_2_ under irradiation. Such dual-functional systems allow for efficient intracellular delivery, photodynamic activation, and ratiometric fluorescence for monitoring therapeutic processes, highlighting the multiple uses of DPBF in advanced biomedical applications [5].

These findings emphasize the need for a critical evaluation of experimental conditions and careful interpretation of results obtained using DPBF, particularly in systems where multiple reactive species may be present [2,4]. A thorough understanding of the photophysical properties of DPBF is critical for its use in fluorescence imaging.

The experimental absorption and fluorescence spectra of DPBF have been reported previously by Zhang and Li [3]. To achieve a deeper understanding of the photophysical properties of DPBF, this study integrates experimental techniques with density functional theory (DFT) and time-dependent DFT (TD-DFT) computational methods in a synergistic manner. Such approaches allow us to better understand the relationship between the structure of DPBF and its photophysical properties, as well as its solvent-dependent behavior. Particular attention is given to evaluating the stability and conformational characteristics of DPBF in both polar aprotic (DMSO and DMF) and protic solvents (ethanol), thus providing a clear insight into how molecular geometry and electronic transitions are influenced by the surrounding solvent environment.

In addition, the intrinsic reaction coordinate (IRC) method was used to explore the reaction mechanism of DPBF and the oxygen molecule. This computational approach established the formation of endoperoxide intermediates and provided a description of the associated transition states. These theoretical findings, supported by experimental data, provide a detailed picture of the reaction mechanism and help clarify how DPBF behaves in its excited state during the interaction with singlet oxygen.

## 2. Results and Discussions

### 2.1. Conformers and Boltzmann Population Analysis

In order to rigorously analyze the possible conformations of DPBF, the relaxed potential energy surface (PES) was investigated at the APFD/3-21G level of theory in the gas phase. The PESs were generated by scanning the two dihedral angles that define the relative orientations of the two phenyl rings with respect to the benzofuran skeleton.

**Figure 1:**
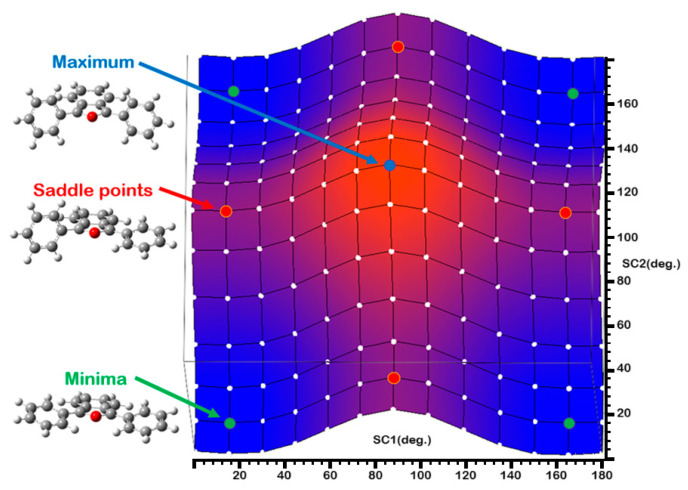
The 2D potential energy surface of the DPBF molecule calculated at the APFD/3-21G level of theory in the gas phase.

The left side of Figure 1 shows the structures for each type of stationary point: minima, saddle points, and the maximum. On the right side, the plot of the 2D-PES illustrates the energy variation as a function of the two scanned dihedral angles.

Nine stationary points were identified. On the 2D-PES, the global minima are indicated with green circles, the global maximum with a yellow circle, and the four transition states with red circles. The four minima are energetically equivalent and correspond to dihedral angle pairs of (15°, 15°), (165°, 165°), (15°, 165°), and (165°, 15°). The structures defined by (15°, 15°) and (165°, 165°) exhibit C_s_ symmetry, while those with (15°, 165°) and (165°, 15°) correspond to C_2_ symmetry. In the case of C_s_ conformers, the reflection plane is perpendicular to the benzofuran skeleton.

The four transition structures are also energetically equivalent and correspond to geometries where one benzene ring forms a 15° angle with respect to the benzofuran plane, while the second ring is oriented at 90°.

### 2.2. Conformational Analysis of the DPBF Molecule

To describe the conformers of the DPBF molecule, all minima and transition states, including the C_2v_ symmetry structures, were re-optimized in the gas phase, ethanol, and dimethyl sulfoxide (DMSO) at the APFD/6-311+G(d,p) level of theory.

Only the C_2_ and C_s_ symmetry structures correspond to true minima, as confirmed by a vibrational frequency analysis. In contrast, the C_2v_ structures exhibit two imaginary frequencies, indicating they are second-order saddle points. These structures are approximately 3 kcal/mol higher in energy and were not considered further.

**Figure 2:**
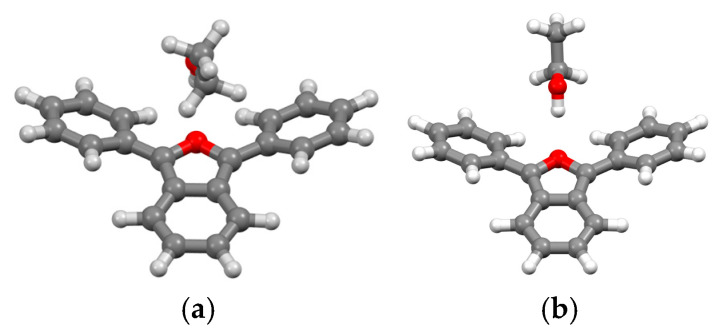
DPBF complex with an ethanol molecule in the c1 (**a**) and c2 (**b**) conformations.

To account for solvent effects, both implicit and explicit solvation models were employed. The explicit model consists of a DPBF–ethanol complex, constructed by adding a single ethanol molecule to the most stable DPBF conformer in two different orientations relative to DPBF (c1 and c2, see Figure 2). The resulting complexes are stabilized through OH…O hydrogen bonding interactions, and subsequently, they were fully re-optimized using the implicit solvation model.

Table 1 summarizes the relative Gibbs free energies (ΔG) and the corresponding Boltzmann populations of the DPBF conformers in the gas phase, ethanol, and DMSO. An analysis of the data shows that, regardless of the solvent, the C_s_ conformer is approximately 0.33–0.4 kcal/mol more stable than the C_2_ conformer.

We primarily employed PCM to account for bulk dielectric effects as a first-order approximation of the solvent environment. This approach was chosen for computational efficiency and because it remains a widely used method for capturing general solvation trends. However, we also considered a hybrid (implicit + explicit) model consisting of a DPBF–ethanol complex embedded in a PCM medium. In this model, an inversion of the relative populations of the c1 and c2 complexes was observed, with both complexes still coexisting in the solution.

Despite the small energy difference, both conformers co-exist at room temperature, with the C_2_ conformer accounting for about half the population of the C_s_ conformer. In the explicit DPBF–ethanol complex, the c1 conformer is 0.25 kcal/mol more stable than c2, corresponding to Boltzmann populations of 60.4% and 39.6%, respectively.

The energies of the DPBF conformers in DMSO were also calculated using the cam-B3LYP/6-311+G(2d,p)//APFD/6-311+G(2d,p) level of theory (Table 2). The results are very similar to those obtained with APFD, with relative populations of 27.31% (C_2_ conformer) and 72.69% (C_s_ conformer). This comparison was carried out to evaluate the ability of the APFD and cam-B3LYP functionals to predict conformer geometries and relative stabilities.

### 2.3. Absorption and Fluorescence Spectra

**Figure 3:**
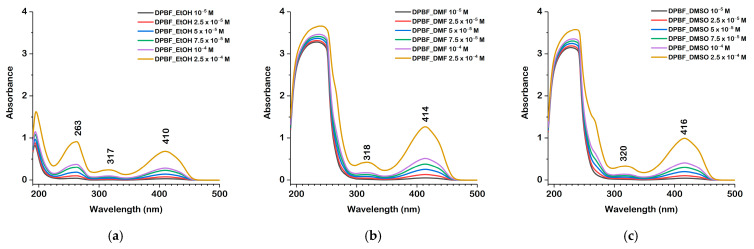
UV-Vis spectra of DPBF in ethanol (**a**), DMF (**b**), and DMSO (**c**) at concentrations ranging from 10^−5^ M to 2.5 × 10^−4^ M.

The UV-Vis spectra of the DPBF were recorded in ethanol, DMF, and DMSO at concentrations ranging from 10^−5^ M to 2.5 × 10^−4^ M, as depicted in Figure 3. The spectra exhibit well-defined absorption maxima, with minor solvent-dependent shifts.

For most concentrations, the absorption maxima appeared at 410 nm in ethanol, 414 nm in DMF, and 416 nm in DMSO. These peaks correspond to the S_0_ → S_1_ electronic transition, which involves a π → π* excitation between the HOMO and LUMO orbitals. The transitions were consistent and clearly resolved at all concentrations. The small shifts towards longer wavelengths observed in DMF and DMSO suggest an enhanced stabilization of the excited state in these aprotic solvents.

The fluorescence excitation and emission spectra measured for DPBF in ethanol, DMF, and DMSO exhibited distinct maxima, indicating predominant radiative transitions.

**Figure 4:**
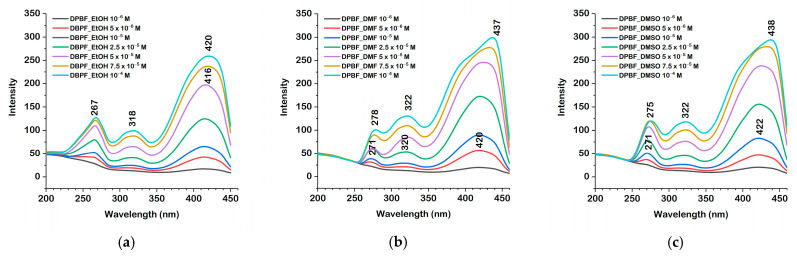
Fluorescence excitation spectra of DPBF in ethanol (**a**), DMF (**b**), and DMSO (**c**) at concentrations ranging from 10^−6^ M to 10^−4^ M.

The excitation spectra of DPBF, as presented in Figure 4, are attributed to π → π* transitions for the S_0_ → S_1_ excitation. The excitation maximum in ethanol occurred at 420 nm, while in DMF and DMSO, it was in the range 420–438 nm. The observed red shift in DMF and DMSO relative to ethanol is consistent with the enhanced stabilization of the excited state in these polar aprotic solvents.

Experimental data show a small, yet consistent blue shift in both the absorption and excitation spectra of DPBF in ethanol relative to DMF and DMSO. This behavior is consistent with the fact that ethanol, while being a protic solvent capable of hydrogen bonding, has a significantly lower dielectric constant than DMF or DMSO. As such, it provides a weaker electrostatic stabilization of the excited state, which is typically more polar. Additionally, specific hydrogen bonding between ethanol and DPBF may preferentially stabilize the ground state, further increasing the energy gap between the ground and excited states. This trend is also observed in the emission spectra, where a slight blue shift in ethanol suggests that excited-state stabilization is indeed less efficient in this solvent. Together, the absorption, excitation, and emission data all support the interpretation that ethanol does not stabilize the excited state as effectively as the polar aprotic solvents, despite its ability to form hydrogen bonds. Our calculations reproduce, to some extent, the experimentally observed blue shift for ethanol, as the PCM model accounts for the solvent’s dielectric constant. However, the slightly larger blue shift observed experimentally can be attributed to the specific solvation characteristics of ethanol, particularly its ability to form hydrogen bonds with DPBF. These interactions likely stabilize the ground state more than the excited state, and the continuum PCM model cannot capture such specific effects.

**Figure 5:**
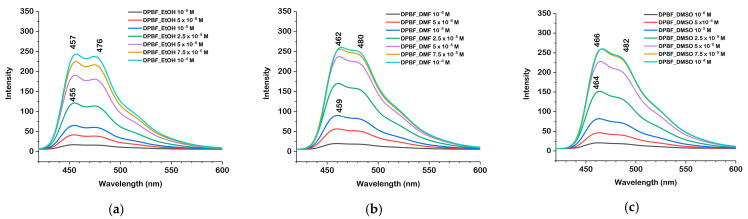
Fluorescence emission spectra of DPBF in ethanol (**a**), DMF (**b**), and DMSO (**c**) at concentrations ranging from 10^−6^ M to 10^−4^ M.

Upon excitation at their absorption maxima, the emission spectra, shown in Figure 5, exhibited structured fluorescence bands. In ethanol, the emission maxima were observed at 455–457 nm, in DMF at 459–462 nm, while in DMSO at 464–466 nm. The emission maxima were recorded upon excitation at 410 nm (ethanol), 414 nm (DMF), or 416 nm (DMSO). The red shift in both the excitation and emission spectra in DMF and DMSO relative to ethanol may be attributed to the greater polarity and aprotic nature of these solutions, which stabilize the excited state more than the ground state, thereby reducing the energy gap and causing the observed shifts.

In addition, excitation and emission spectra were recorded at a higher concentration (2.5 × 10^−4^ M), in all solvents. At this concentration, significant changes were observed in both spectra of the DPBF. Figures S1 and S2 show the excitation and emission spectra of the DPBF recorded in ethanol, DMF, and DMSO at two concentrations: 10^−4^ M and 2.5 × 10^−4^ M.

Compared to the spectra recorded at 10^−4^ M, the excitation bands are significantly affected. The main absorption peak is red-shifted by more than 10 nm in all the cases, and several new peaks appear at shorter wavelengths. The intensity of the emission bands (Figure S2) is reduced, particularly in the case of the DMF and DMSO solvents, and the emission peaks exhibit a slight red shift.

These effects can be attributed to a combination of processes. At higher concentrations, the primary inner filter effect becomes significant, as the excitation light is mostly absorbed at the front of the cuvette, resulting in less light reaching deeper into the sample and, thus, a distortion of the excitation spectrum. Additionally, at this concentration, DPBF molecules may begin to aggregate in the ground state through π → π interactions, leading to the formation of new absorbing species and the appearance of additional peaks in the excitation spectra at shorter wavelengths.

The secondary inner filter effect, caused by a spectral overlap between absorption and emission, further reduces fluorescence intensity and causes a slight red shift of the emission maxima. Furthermore, the decreased emission intensity can also be attributed to self-quenching, a process in which excited DPBF molecules interact with each other in a solution, leading to non-radiative decay and, consequently, diminished fluorescence.

**Figure 6:**
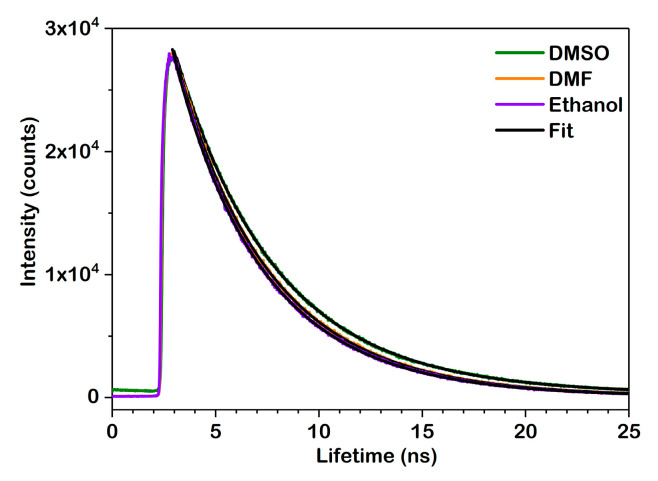
Fluorescence lifetime decay curves of DPBF in ethanol, DMF, and DMSO with corresponding fit model.

The fluorescence lifetimes ($\tau_{f}$) of DPBF in ethanol, DMF, and DMSO were measured at a concentration of 10^−4^ M and are shown in Figure 6, where the experimental decay curves are fitted with a single-exponential function. Time-resolved fluorescence measurements revealed a single-exponential decay profile for DPBF in all the solvents studied. The fluorescence lifetimes were 4.6240 ± 0.00027 ns in ethanol, 4.7140 ± 0.00035 ns in DMF, and 4.9100 ± 0.00032 ns in DMSO.

The progressive increase in fluorescence lifetime with solvent polarity is consistent with the red shift observed in the emission spectra, confirming the enhanced stabilization of the excited state in more polar environments.

To further evaluate the solvent effect on the absorption intensity, the molar absorption coefficients (ε) of DPBF were calculated using the linear regression of absorbance and concentration (Figure S3). The calculated values are approximately 2.71 × 10^3^ M^−1^ · cm^−1^ in ethanol, 3.95 × 10^3^ M^−1^ · cm^−1^ in DMSO, and 5.06 × 10^3^ M^−1^ · cm^−1^ in DMF, with corresponding lg ε values of 3.43, 3.60, and 3.70. These values show a moderate dependence on the solvent used and are slightly lower than those reported in reference [3], where the calculated lg ε values were 4.39 in ethanol and 4.36 in DMSO/DMF.

The lower values in ethanol can be explained by its protic nature and ability to form hydrogen bonds, which can slightly alter the excited state. This interpretation is supported by the reduced fluorescence lifetime in ethanol (4.61 ns) compared to aprotic solvents (4.71 ns and 4.91 ns). TD-DFT calculations also yielded a slightly lower oscillator strength (f) and transition dipole moment (|μ_10_|^2^) in ethanol, indicating that hydrogen bonding may reduce the radiative transition probability.

The geometries of DPBF in its ground and excited states, optimized at the APFD/6-311+G(2d,p) level in DMSO, are shown in Figure S4. The comparison of the two geometries reveals that, upon excitation, the molecule undergoes a slight transverse elongation (along the axis joining the benzene rings) and a slight longitudinal compression (along the bisector of the benzofuran core). The greatest differences are seen in the bond lengths highlighted by ellipses in Figure S4. Red ellipses indicate bond shortening, while blue ellipses indicate bond elongation in the excited state.

Overall, the excitation induces significant changes in the planarity and characteristic bond parameters of the benzofuran skeleton.

### 2.4. Photophysical Parameters of the DPBF Molecule

The energies corresponding to the electronic absorption and emission transitions, as well as the fluorescence lifetime, were calculated using the TD-DFT method. Table 3 summarizes the results obtained in this study.

The DPBF experimental absorption spectra showed distinct maxima in the 410–416 nm range, with minor shifts depending on the solvent polarity. Among the tested functionals, cam-B3LYP predicted absorption maxima between 397 and 398.3 nm, underestimating the experimental values by approximately 15 nm, yet demonstrating the highest accuracy overall. The ωB97X-D functional provided reasonably accurate predictions (389.2–390.5 nm), underestimating the experimental values by approximately 20–25 nm. In contrast, the LC-ωHPBE functional significantly underestimated the absorption maxima by more than 50 nm, while the B3LYP, APFD, and PBE0 functionals overestimated the experimental values by approximately 25–40 nm.

The experimentally measured fluorescence emission maxima for DPBF varied slightly with the solvent polarity and ranged from 455 to 466 nm. The calculated maxima in the 470.1–471.8 nm range closely matched the experimental values, indicating that the ωB97X-D functional best reproduced the vertical and adiabatic emission energies (λ_em_). Additionally, the cam-B3LYP functional showed good agreement (478.7–480.4 nm) and moderate deviations (15–25 nm). However, the B3LYP, APFD, and PBE0 functionals overestimated the emission maxima by more than 50 nm, while LC wHPBE significantly underestimated them, predicting values in the range of 431.8 to 433.2 nm, approximately 35 to 40 nm below the experimental results.

The calculated oscillator strengths (f) were comparable across all functionals, ranging from approximately 0.57 to 0.65. The highest values were obtained using LC-ωHPBE (0.6338–0.6472), followed by cam-B3LYP (0.6101–0.6229) and ωB97X-D (0.6050–0.6188). In contrast, APFD, B3LYP, and PBE0 yielded slightly lower oscillator strengths, with values between 0.565 and 0.590.

Fluorescence lifetimes (τ_f_) for DPBF that were measured experimentally varied from 4.62 ns for ethanol to 4.91 ns for DMSO, and they slightly increased with solvent polarity. Theoretical predictions and experimental data were in good agreement when using cam-B3LYP (5.65–5.67 ns) and particularly ωB97X-D (5.48–5.49 ns). In contrast, functionals like B3LYP and APFD significantly overestimated the radiative lifetimes (τ_r_), predicting values between 6.55 and 7.04.

It is worth mentioning that, as shown in Table 3, the APFD functional outperforms B3LYP in reproducing experimental transition energies, although its accuracy remains slightly inferior to that of PBE0. Owing to its improved performance, APFD has increasingly been employed in the calculation of transition energies for various molecular systems [6,7,8,9,10].

The range-separated cam-B3LYP functional is widely utilized in time-dependent density functional theory (TD-DFT) calculations for predicting the absorption and emission energies of molecules. It is often preferred over PBE0 or B3LYP for systems where the long-range electron correlation effects are significant, as it incorporates an explicit correction for such interactions [11,12,13,14,15,16].

Concluding, when combining theoretical and experimental methods, the functionals cam-B3LYP and ωB97X-D provide the best overall approximation of the spectroscopic and photophysical parameters of DPBF. This conclusion is supported by the data presented in Table 4, which reports the mean absolute errors (MAE) and root mean squared errors (RMSE), in nanometers, for all the density functionals employed. It is also worth noting that both functionals have previously demonstrated excellent performance in predicting the excited-state properties of difluoroboranes and hydroxyphenylimidazo[1,2-a]pyridine derivative fluorescent dyes [17].

We would like to emphasize that the addition of an ethanol molecule in the c1 and c2 complexes does not significantly affect the calculated λ_max_. For example, at the cam-B3LYP/6-311+G(2d,p) level of theory, the calculated λ_max_ for the c1 complex is 393.4 nm—only 3.6 nm shorter than that of the isolated DPBF molecule (see Table S1). The λ_max_ values calculated for the c2 complex, using all tested functionals, remain close to those of c1 within a 2 nm range.

Interestingly, when geometry optimizations are performed using the APFD functional, and the UV-Vis spectrum is subsequently calculated using the cam-B3LYP functional on the APFD-optimized geometry [cam-B3LYP/6-311+G(2d,p)//APFD/6-311+G(2d,p) level of theory], the resulting λ_max_ value in ethanol (411.7 nm) is in excellent quantitative agreement with the experimental data (410.0 nm). A similar behavior is also observed in the case of the ωB97XD functional.

### 2.5. Electronic Structure and Nature of the Transition

**Figure 7:**
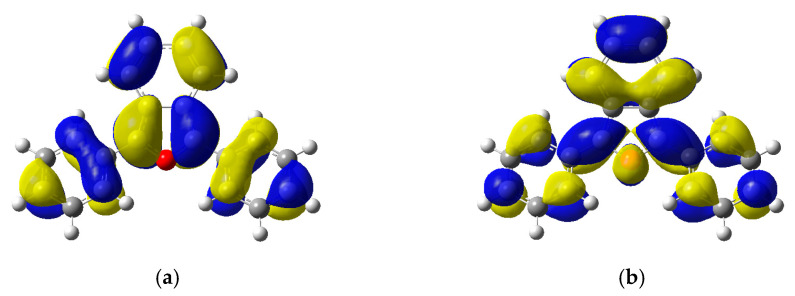
Natural transition orbitals HOTO (**a**) and LUTO (**b**) calculated at the cam-B3LYP/6-311+G(2d,p) level of theory in DMSO (surface isovalue 0.04 a.u.).

The nature of the excited states has been analyzed using the Natural Transition Orbitals (NTO) formalism proposed by Martin [18]. Figure 7 depicts the hole (HOTO) and particle (LUTO) natural transition orbitals (NTOs) involved in the transition, corresponding to λ_max_ of DPBF. A π → π* transition between the HOTO and LUTO is confirmed by an orbital coefficient of 0.9906, showing that the excitation is characterized by a single configuration with a 99.06% contribution.

### 2.6. Reaction Mechanism

To elucidate the reaction mechanism, the proposed pathway was investigated using DFT calculations. Reactions of singlet oxygen with furans generally proceed through the formation of a 2,5-endoperoxide, as is the case here.

**Figure 8:**
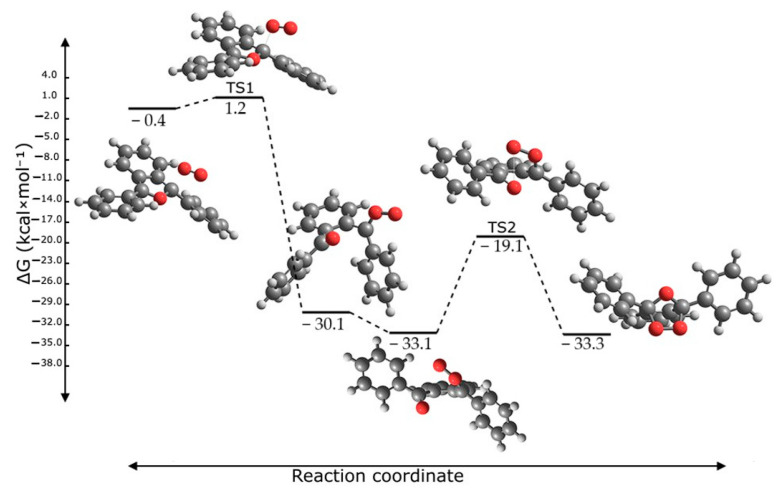
Calculated reaction profile in DMF solvent, and the structures of the corresponding reactant complexes, transition states, and product.

The stability of the various species was determined by calculating their Gibbs free energy in solution, which was then used to construct the reaction profiles. Figure 8 illustrates the structures of the reactant complexes, transition states, and products of the pathways studied in DMF solvent. The profiles in the other solvents are very similar and are depicted in Figure S5. In the present mechanism, the reaction is initiated by the formation of an exciplex (eximer) with the electronically excited ^1^O_2_, which forms an adduct with the DPBF molecule in the ground state. Through a small barrier of less than 1 kcal/mol, the oxygen bonds to the C5 furan carbon, leading to the opening of the furan ring and forming a zwitterion intermediate. This step is highly exoergic.

The zwitterionic intermediate presents a partial charge of −0.56 e on the peroxy group oxygen, −0.54 e on the carbonyl oxygen, 0.38 e on the carbonyl carbon, and 0.44 e on the peroxy-bonding carbon. For the second step, the zwitterionic intermediate undergoes internal rotation, resulting in a new geometry that promptly reacts through the simultaneous bonding of the terminal oxygens to the C2 and C5 atoms, forming the closed product structure. This transformation, which involves a barrier height of 14 kcal/mol, constitutes the rate-limiting step of the mechanism. The final product is only marginally more stable than the zwitterionic intermediate.

**Figure 9:**
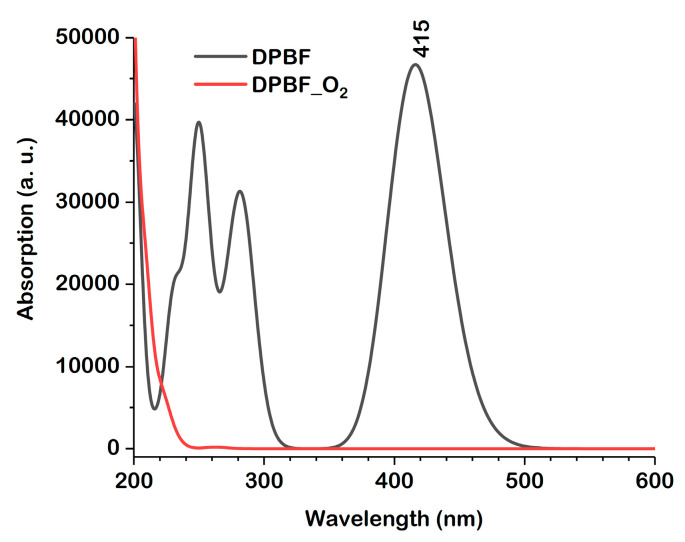
Calculated UV-VIS spectra of DPBF and the 2,5-endoperoxid in DMF at the cam-b3lyp/6-311+G(d,p) level.

DPBF can quantitatively react with ^1^O_2_, disrupting the π system of isobenzofuran, which prevents the product from absorbing or emitting visible light, ultimately leading to the formation of o-dibenzoylbenzene. Figure 9 displays the calculated UV–Vis spectra of the DPBF and the 2,5-endoperoxide. For DPBF, the absorption maximum corresponding to the S_0_ → S_1_ electronic transition is located at 415 nm, which matches excellently with the experimental value of 415 nm [3] and shows minimal dependence on the solvent used. In contrast, the spectrum of the 2,5-endoperoxide is entirely different, exhibiting absorption only at high energies. The lowest-energy transition is complex and displays very low intensity (Table 4).

**Figure 10:**
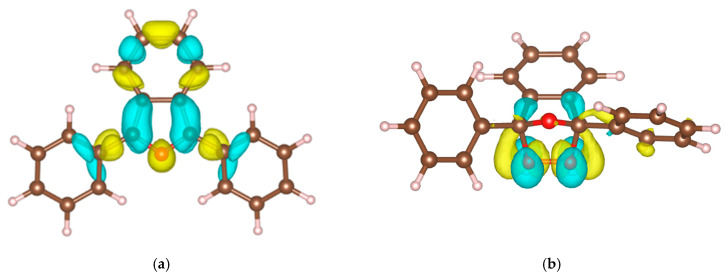
Contour plots of the electron density difference (Δρ) for the lowest-energy excitation of DPBF (**a**) and the 2,5-endoperoxide (**b**). Yellow denotes a positive contribution (increase in electron density), and blue indicates a reduction of electron density (Table 5).

Figure 10 illustrates the electron density differences associated with the lowest-energy transitions of DPBF and the 2,5-endoperoxide, revealing the regions of the molecules that lose or gain electrons upon excitation. While the transition corresponding to DPBF exhibits a clear π → π* character, the analogous transition of the 2,5-endoperoxide appears to have an n → π* character.

**Table 5:** Lowest energy transitions, oscillator strengths, and molecular contributions of DPBF, the 2,5-endoperoxide at the cam-B3LYP/6-311++g(d,p) level of theory in DMF solvent. The first 2,5-endoperoxide excitation with significant intensity is also displayed.

**DPBF in DMF**			
State	λ (nm)	*f*	Major MO → MO Contributions
S_1_	415.9	0.6445	HOMO → LUMO (98%)
S_2_	282.6	0.2454	HOMO → L + 1 (89%)
S_3_	282.3	0.1528	HOMO → L + 3 (86%), HOMO → L + 2 (6%), HOMO → L + 5 (2%)
**2,5-endoperoxide in DMF**			
State	λ (nm)	*f*	Major MO → MO contributions
S_1_	262.3	0.0026	H-6 → LUMO (18%), H-6 → L + 13 (19%), H-1 → LUMO (14%) H-6 → L + 4 (3%), H-4 → LUMO (2%), H-3 → LUMO (2%), H-2 → LUMO (3%), H-1 → L + 13 (8%)
S_2_	234.1	0.0004	H-1 → LUMO (16%), HOMO → L + 2 (31%), H-6 → LUMO (7%), H-5 → L + 2 (2%), H-4 → L + 2 (3%), H-1 → L + 1 (3%), H-1 → L + 3 (2%), H-1 → L + 4 (3%), H-1 → L + 6 (5%), HOMO → L + 1 (4%), HOMO → L + 4 (2%)
S_3_	230.1	0.0084	H-3 → L + 1 (12%), H-2 → L + 1 (18%), H-5 → L + 2 (2%), H-5 → L + 5 (6%), H-5 → L + 6 (5%), H-4 → LUMO (4%), H-4 → L + 1 (7%), H-4 → L + 2 (6%), H-4 → L + 5 (4%), H-4 → L + 6 (4%), H-3 → L + 5 (6%), H-3 → L + 6 (2%), H-2 → LUMO (7%), H-1 → L + 1 (2%)
S_6_	208.1	0.1825	H-5 → L + 1 (11%), H-4 → LUMO (13%), H-3 → LUMO (11%), H-1 → LUMO (14%), H-5 → LUMO (5%), H-3 → L + 4 (3%), H-3 → L + 6 (5%), H-2 → L + 2 (3%), H-2 → L + 4 (3%), HOMO → LUMO (3%), HOMO → L + 5 (4%)

## 3. Experimental Details

1,3-Diphenylisobenzofuran (DPBF) was purchased from Merck and used as received. The photophysical properties of DPBF have been investigated in ethanol, dimethylformamide (DMF), and dimethyl sulfoxide (DMSO). Solutions were prepared at concentrations ranging from 10^−6^ M to 2.5 × 10^−4^ M for detailed spectroscopic investigations. All experiments were carried out at room temperature.

UV-Vis spectra have been recorded using a Jasco V-630 double-beam UV-Vis spectrophotometer (Hachioji, Japan) equipped with a silicon photodiode detector. Excitation and emission spectra were recorded with a Jasco FP-6500 spectrofluorometer. Emission spectra were measured following excitation at 410 nm for ethanol, 414 nm for DMF, and 416 nm for DMSO, with respective spectral ranges of 415–600 nm, 419–600 nm, and 421–600 nm. Excitation spectra were measured by monitoring the emission at wavelengths of 456 nm (ethanol), 462 nm (DMF), and 465 nm (DMSO), over spectral ranges of 200–450 nm for ethanol and 200–460 nm for DMF and DMSO. All fluorescence measurements were conducted with a spectral resolution of 1 nm.

Fluorescence lifetime measurements were performed using a MicroTime200 time-resolved confocal fluorescence microscope system from PicoQuant (Berlin, Germany). The excitation source was a 405 nm diode laser (LDH-D-C-405, 40 MHz, 0.07 to 1 µW at the probe site, PicoQuant). The instrument response function (IRF) was recorded by measuring the backscattered laser light from a glass coverslip. Fluorescence decays were recorded and analyzed using a monoexponential fit. In order to ensure accurate lifetime determination, the fitting process accounted for background counts, IRF contributions, and shifts between the IRF and decay profiles.

## 4. Computational Methods

Density functional theory (DFT) and time-dependent DFT (TD-DFT) methods have been used to investigate the photophysical properties of the DPBF molecule [19]. Geometry optimizations, normal modes analysis, and electronic transition calculations were performed using the APFD [20], B3LYP [21,22,23,24] cam-B3LYP [25], PBE0 [26], LC-ωHPBE [27], and ωB97X-D [28] functionals coupled with the 6-31G(d), 6-311+G(d,p), and 6-311+G(2d,p) basis sets [6]. The APFD functional has been tested in this study due to its good performance in describing the potential energy surfaces and relative conformational energies [20].

Solvent effects were taken into account using the Polarizable Continuum Model (PCM) [29], considering ethanol, dimethyl sulfoxide (DMSO), and dimethylformamide (DMF) as solvents.

For the calculation of relative Boltzmann populations, the following equation was used:

(1) $$P_{i}=g_{i}\frac{e^{\left(-\frac{∆G_{i}}{k_{B}T}\right)\;}}{\sum_{i}g_{i}e^{\left(-\frac{∆G_{i}}{k_{B}T}\right)\;}}$$

where ΔG_i_ are the relative free energies (Gibbs), k_B_ is the Boltzmann constant, T is the temperature in Kelvin degrees (298.15 K), and g_i_ is the degree of degeneracy for each conformer (g_i_ = 2 for conformers with C_1_ symmetry and g_i_ = 1 for conformers with C_2_ or C_i_ symmetry) [30,31,32].

Tight and very tight criteria were used for the convergence of the molecular geometries and SCF calculations, respectively. The ultrafine grid was employed for the numerical integration of the electronic density.

Frequency calculations confirmed that all of the optimized structures, both gas phase and solution, correspond to true minima on the potential energy surface. For IRC, transition states were located as first-order saddle points on the potential energy surface and identified by a single imaginary vibrational frequency. The connectivity between transition states and their associated reactants and products was established by intrinsic reaction coordinate (IRC) calculations, confirming the reaction pathways.

Atomic charges were calculated by fitting the molecular electrostatic potential using the CHELPG scheme proposed by Breneman and Wiberg [33].

All calculations were performed using Gaussian 16, Revision C.01 [34]. GaussView 6.1.1 [35] was used to plot molecular orbitals and electronic density differences.

## 5. Conclusions

We have conducted a comprehensive investigation, combining experimental and theoretical approaches, to study the spectroscopic, photophysical, and reactive properties of 1,3-diphenylisobenzofuran (DPBF) in polar protic (ethanol) and aprotic (DMSO, DMF) solvents. This combined approach provided a comprehensive understanding of the behavior of this crucial fluorescent probe for singlet oxygen.

A conformational analysis for DPBF itself revealed the co-existence of two stable conformers, C_2_ and C_s_, with the C_s_ conformer being consistently more stable by 0.33–0.40 kcal·mol^−1^ at room temperature across all studied solvents. When one ethanol molecule was explicitly added to form hydrogen-bonded complexes, the c1 conformer was more stable than c2 by 0.25 kcal·mol^−1^, resulting in Boltzmann populations of 60.4% (c1) and 39.6% (c2).

Spectroscopic investigations using UV-Vis and fluorescence measurements confirmed a well-resolved S_0_ → S_1_ electronic transition of a π → π* nature with an absorption maxima between 410 and 416 nm, from ethanol to DMSO. The emission maxima ranged from 455 to 466 nm, going from ethanol to DMSO. The fluorescence lifetimes also increased with the solvent polarity, with the values being 4.62 ns, 4.71 ns, and 4.91 ns for ethanol, DMF, and DMSO, respectively. A consistent red shift was observed in both the absorption and emission spectra in the polar aprotic solvents, which, together with a gradual increase in fluorescence lifetimes with solvent polarity, indicates an enhanced stabilization of the excited state in these environments.

The photophysical parameters were obtained by means of TD-DFT calculations using several functionals. Overall, the ωB97X-D and cam-B3LYP functionals provided the best agreement with the experimental data for absorption/emission wavelengths and fluorescence lifetimes. Cam-B3LYP yielded absorption maxima around 397–398 nm and emission maxima at 478–480 nm, with radiative lifetimes of ~5.67 ns. ωB97X-D predicted slightly lower absorption (389–391 nm) and emission maxima (470–472 nm), with lifetimes of ~5.48 ns. Mean absolute errors (MAE) in λ_max_ predictions further confirmed the superior accuracy of cam-B3LYP (MAE = 15.5 nm) and ωB97X-D (MAE = 23.4 nm).

Furthermore, the study revealed that the reaction mechanism is a two-step pathway with a low energy barrier. This pathway begins with the formation of a zwitterionic intermediate. The reaction then goes through a second, rate-limiting step with a 14 kcal·mol^−1^ energy barrier, resulting in the final 2,5-endoperoxide product. The calculated absorption spectrum of this endoperoxide shows no absorption in the visible range, explaining the fluorescence loss in the interaction of DPBF with ^1^O_2_.

## Figures and Tables

**Table 1:** Relative Gibbs free energies and Boltzmann populations of DPBF conformers calculated at the APFD/6-311+G(2d,p) level of theory.

Conformer	ΔG (kcal/mol)	The Boltzmann Factor	Relative Boltzmann Population (%)
DPBF in gas phase			
C_2_	0.33	0.573	36.42
C_s_	0.00	1.000	63.58
DPBF in ethanol			
C_2_	0.40	0.509	33.74
C_s_	0.00	1.000	66.26
DPBF in DMSO			
C_2_	0.40	0.509	33.74
C_s_	0.00	1.000	66.26
DPBF with an ethanol molecule in ethanol			
c1	0.00	1.000	60.40
c2	0.25	0.656	39.60

**Table 2:** Relative Gibbs free energies and Boltzmann populations of DPBF conformers calculated at the cam-B3LYP/6-311+G(2d,p)//APFD/6-311+G(2d,p) level of theory.

Conformer	ΔG (kcal/mol)	The Boltzmann Factor	Relative Boltzmann Population (%)
DPBF in DMSO			
C_2_	0.58	0.376	27.31
C_s_	0.00	1.000	72.69

**Table 3:** Calculated absorption and emission energies, oscillator strengths, and radiative lifetimes of DPBF at the APFD, B3LYP, cam-B3LYP, PBE0, ωB97X-D, and LC-ωHPBE levels of theory (6-311+G(2d,p) basis set).

Basis Set	Functional	Solvent	λ_abs_ (nm)	f (a.u.)	λ_em_ (nm) Vertical/Adiabatic	|μ_10_|^2^ (a.u.)	τ_r_ (ns)
6-311+G(2d,p)	APFD	EtOH	440.1	0.5735	568.6/516.5	13.7952	6.58
		DMF	441.9	0.5882	570.3/517.8	13.9729	6.55
		DMSO	441.5	0.5856	571.0/518.3	14.0475	6.55
	B3LYP	EtOH	451.1	0.5652	583.6/530.6	13.9355	7.04
		DMF	452.8	0.5778	585.4/532.0	14.1166	7.01
		DMSO	452.4	0.5752	586.1/532.5	14.1926	7.00
	cam-B3LYP	EtOH	397.0	0.6101	544.2/478.7	14.0328	5.67
		DMF	398.3	0.6229	545.9/480.0	14.1974	5.66
		DMSO	398.2	0.6214	546.7/480.4	14.2664	5.65
	PBE0	EtOH	436.4	0.5807	564.5/512.5	13.8025	6.43
		DMF	439.4	0.5935	566.2/515.8	13.9781	6.41
		DMSO	438.5	0.5911	566.9/514.4	14.0517	6.40
	ωB97X-D	EtOH	389.2	0.6050	536.8/470.1	13.9017	5.49
		DMF	390.5	0.6188	538.5/471.2	14.0644	5.48
		DMSO	390.1	0.6149	539.3/471.8	14.1327	5.48
	LC-ωHPBE	EtOH	351.7	0.6338	508.0/431.8	13.6037	4.76
		DMF	352.8	0.6472	509.7/432.8	13.7533	4.75
		DMSO	352.6	0.6448	510.4/433.2	13.8161	4.75

**Table 4:** Performance of the used density functionals for predicting λ_max_ for DPBF. Errors are reported as mean absolute errors (MAE) and root mean squared errors (RMSE) in nm.

Functional	Δλ (nm) EtOH	Δλ (nm) DMF	Δλ (nm) DMSO	MAE (nm)	RMSE (nm)
cam-B3LYP	13.0	15.7	17.8	15.50	15.62
ωB97X-D	20.8	23.5	25.9	23.40	23.49
PBE0	26.4	25.4	22.5	24.77	24.82
APFD	30.1	27.9	25.5	27.83	27.90
B3LYP	41.1	38.8	36.4	38.77	38.81
LC-ωHPBE	58.3	61.2	63.4	60.97	61.00

## Data Availability

The original contributions presented in this study are included in the article/Supplementary Material. Further inquiries can be directed to the corresponding author.

## Supplementary Materials

The following supporting information can be downloaded at https://www.mdpi.com/article/10.3390/molecules30143021/s1, Figure S1: Fluorescence excitation spectra of DPBF in ethanol (a), DMF (b), and DMSO (c) recorded at concentrations of 10^−4^ M and 2.5 × 10^−4^ M.; Figure S2: Fluorescence emission spectra of DPBF in ethanol (a), DMF (b), and DMSO (c) recorded at concentrations of 10^−4^ M and 2.5 × 10^−4^ M.; Figure S3. Linear correlation between absorbance and concentration for DPBF in ethanol, DMF, and DMSO. Figure S4: The bond length of the DPBF molecule in the ground and excited states, obtained at the APFD/6-311+G(2d,p) level of theory. Figure S5: Calculated reaction profile of DPBF in DMF, DMSO, and ethanol solvents. Table S1: Performance of the used density functionals for predicting λ_max_ for DPBF. Errors are reported as mean absolute errors (MAE) and root mean squared errors (RMSE) in nm.
