# Supplementary material for: Photophysical Properties of 1,3-Diphenylisobenzofuran as a Sensitizer and Its Reaction with O2
Source: Molecules. 2025 Jul 18;30(14):3021. doi: 10.3390/molecules30143021 (PMC12298378; doi:10.3390/molecules30143021)
Supplement: Supplementary file 1 [file molecules-30-03021-s001.zip › molecules-3754308-supplementary.pdf]

Supplementary material

Photophysical Properties of 1,3-Diphenylisobenzofuran as a Sensitizer and Its Reaction with O_2

Ștefan Stan[1], JoãoP. Prates Ramalho[2,3] Alexandru Holca[1,4] and Vasile Chiș[1*]

Faculty of Physics, Babes-Bolyai University, Str. M. Kogălniceanu 1, RO-400084 Cluj-Napoca, Romania; stefan.stan@ubbcluj.ro (Ș.S.); alexandru.holca@ubbcluj.ro (A.H.)

2 Department of Chemistry and Biochemistry, School of Science and Technology, University of Évora, Rua Romão Ramalho 59, 7000-671 Évora, Portugal; jpcar@uevora.pt

3 Hercules Laboratory, University of Évora, Palácio do Vimioso, Largo Marquês de Marialva 8, 7000-809 Évora, Portugal

4 Nanobiophotonics and Laser Microspectroscopy Center, Interdisciplinary Research Institute in Bio-Nano-Sciences, Babes-Bolyai University, T. Laurian 42, RO-400271 Cluj-Napoca, Romania

* Correspondence: vasile.chis@ubbcluj.ro

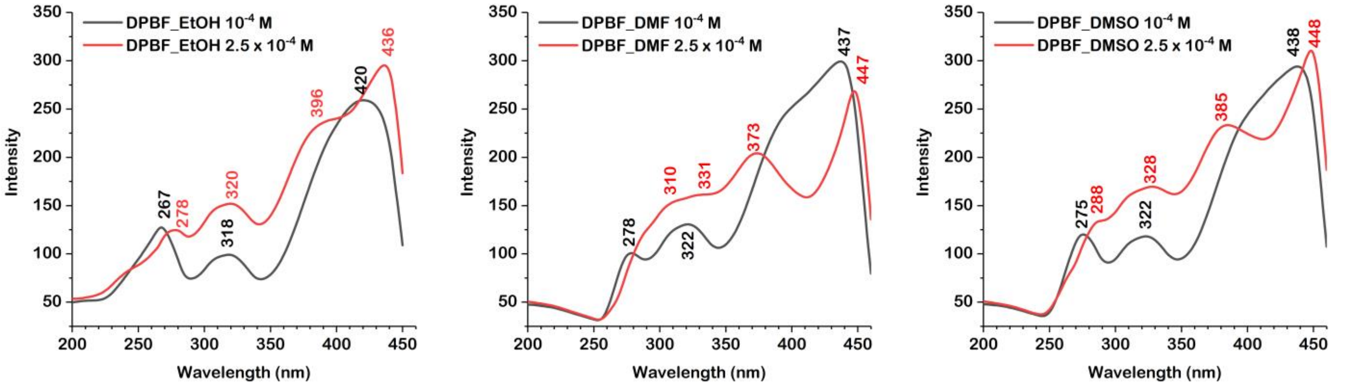

(a) (b) (c)

Figure S1. Fluorescence excitation spectra of DPBF in ethanol (a), DMF (b), and DMSO (c) recorded at concentrations of 10^{-4} M and $2.5 \cdot 10^{-4}$ M.

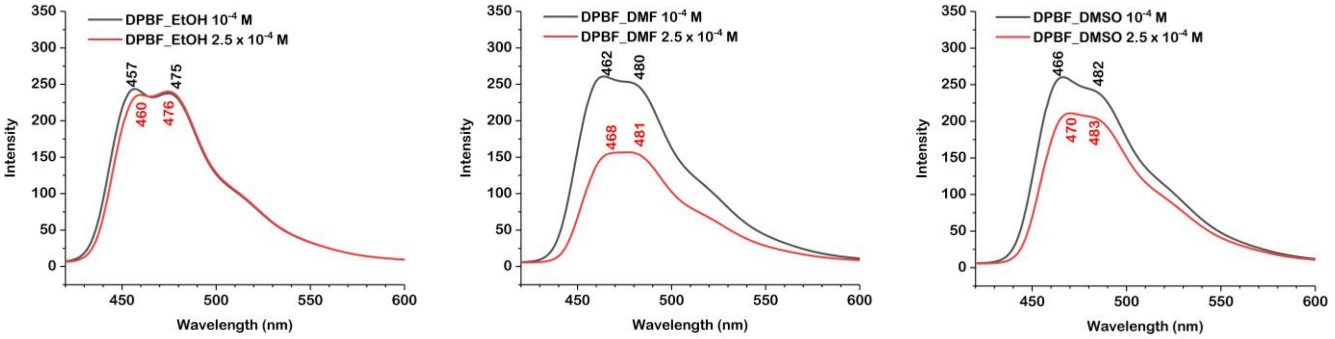

(a) (b) (c)

Figure S2. Fluorescence emission spectra of DPBF in ethanol (a), DMF (b), and DMSO (c) recorded at concentrations of 10^{-4} M and $2.5 \cdot 10^{-4}$ M.

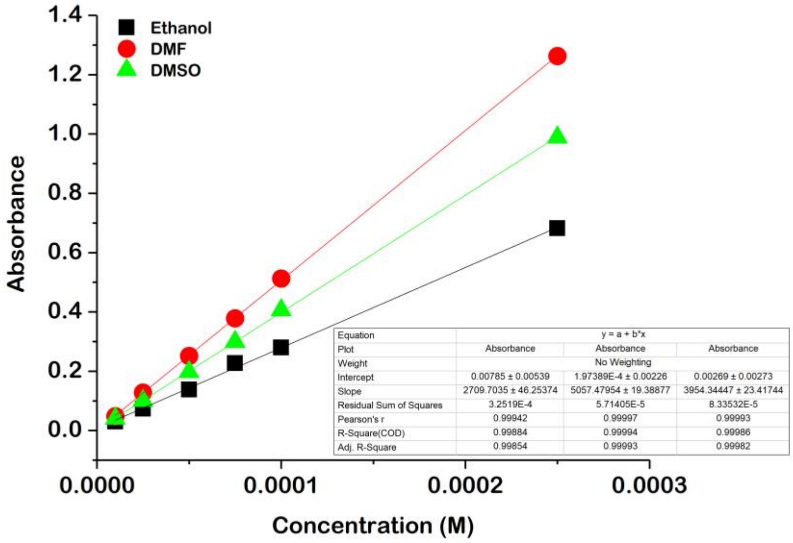

Equation	y = a + b*x		
Plot	Absorbance	Absorbance	Absorbance
Weight	No Weighting		
Intercept	0.00785 ± 0.00539	1.97389E-4 ± 0.00226	0.00269 ± 0.00273
Slope	2709.7035 ± 46.25374	5057.47954 ± 19.38877	3954.34447 ± 23.41744
Residual Sum of Squares	3.2519E-4	5.71405E-5	8.33532E-5
Pearson's r	0.99942	0.99997	0.99993
R-Square(COD)	0.99884	0.99994	0.99986
Adj. R-Square	0.99854	0.99993	0.99982

Figure S3. Linear correlation between absorbance and concentration for DPBF in ethanol, DMF, and DMSO.

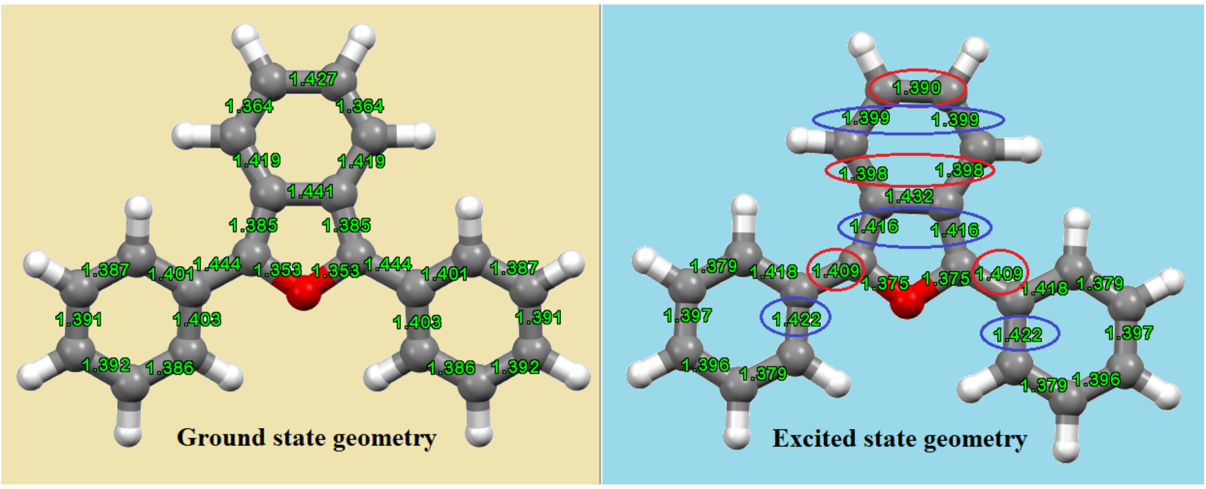

Figure S4. The bond length of the DPBF molecule in the ground and excited states, obtained at the APFD/6-311+G(2d,p) level of theory.

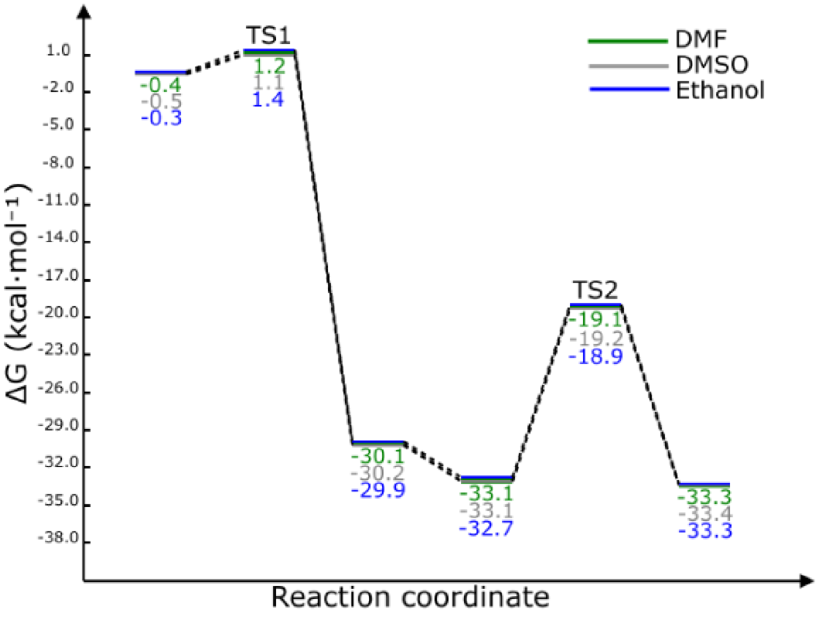

Figure S5. Calculated reaction profile of DPBF in DMF, DMSO and ethanol solvents.

Table S1. Comparison of the calculated λ_{max} values for the c1 and c2 complexes in EtOH relative to the DPBF molecule.

Level of theory	System		
	DPBF	c1	c2
cam-B3LYP/6-311+G(2d,p)	397.0	393.4	393.3
cam-B3LYP/6-311+G(2d,p)//APFD/6-311+G(2d,p)	-	411.7	409.7
wB97X-D/6-311+G(2d,p)	389.2	388.2	388.0
wB97X-D/6-311+G(2d,p)//APFD/6-311+G(2d,p)	-	406.8	405.1
B3LYP/6-311+G(2d,p)	451.1	448.4	447.5
B3LYP/6-311+G(2d,p)//APFD/6-311+G(2d,p)	-	448.1	446.1
PBE0/6-311+G(2d,p)	436.4	433.4	433.6
PBE0/6-311+G(2d,p)//APFD/6-311+G(2d,p)	-	436.9	434.9
APFD/6-311+G(2d,p)//APFD/6-311+G(2d,p)	440.1	439.6	437.7
